# Epidemiology and clinicopathological features of soft tissue tumors in adolescents: a cross-sectional study

**DOI:** 10.1186/s12887-024-05361-2

**Published:** 2025-01-09

**Authors:** Ogochukwu Chioma Ofiaeli, Felix Emeka Menkiti, Victor Ifeanyichukwu Modekwe, Shirley Nneka Chukwurah, Ogochukwu Robinson Ofiaeli, Amalachukwu Okwukweka Odita

**Affiliations:** 1 Department of Paediatrics, Nnamdi Azikiwe University, Nnamdi Azikiwe University Teaching Hospital, Nnewi, Anambra state Nigeria; 2 Department of Anatomic Pathology and Forensic Medicine/Histopathology, Nnamdi Azikiwe University, Nnamdi Azikiwe University Teaching Hospital, Nnewi, Anambra state Nigeria; 3 Department of Surgery, Nnamdi Azikiwe University, Nnamdi Azikiwe University Teaching Hospital, Nnewi, Anambra State Nigeria; 4 Department of Medicine, Nnamdi Azikiwe University, Nnamdi Azikiwe University Teaching Hospital, Nnewi, Anambra State Nigeria; 5 Department of Medical Microbiology, Nnamdi Azikiwe University, Nnamdi Azikiwe University Teaching Hospital, Nnewi, Anambra State Nigeria

**Keywords:** Soft tissue neoplasms, Adolescents, Histopathology, Tertiary care center, Patterns

## Abstract

**Background:**

Soft tissue tumors (STTs) in adolescents are relatively rare, and their characteristics and behavior have not been well studied in this age group. The aim of this study was to describe the clinicopathologic patterns of STTs in adolescents aged 10–19 years according to the 2020 WHO classification.

**Method:**

A 10-year retrospective cross-sectional study of 632 surgical samples from adolescents was conducted at a tertiary health facility to determine the frequency, histological patterns and characteristics of STTs in this population. The data were analyzed via SPSS 23.

**Results:**

STTs accounted for 12.5% of all histologically diagnosed lesions in adolescents, with a mean age of 15 ± 2.9 years, 54.4% occurring in females and 35.4% in middle adolescents. The majority (64.56%) of STTs were benign, while malignant and intermediate-grade neoplasms accounted for 25.32% and 10.13%, respectively. Vascular tumours were the most common, followed by adipocytic and fibroblastic/myofibroblastic tumours, with hemangiomas being the most common. The most prevalent symptom was a painless mass (82.3%), with the head and neck (25.3%) being the most commonly involved body site. Most of the STTs patients presented within the first two years of occurrence (36.7%, *n* = 29/79). However, age, age group and sex were not significantly associated with the WHO grades of these STTs.

**Conclusion:**

This study provided valuable insights into the characteristics and behavior of STTs in adolescents, highlighting the importance of early diagnosis and management. These findings suggest that adolescent STTs affect females more than males , involve the head and neck more and are more benign, with vascular tumours being the most common type of STT in this age group.

## Background

Mesenchymal neoplasms (MTs)/ soft tissue tumors (STTs) represent a diverse array of tumors originating from precursor cells derived from the mesoderm, which differentiate into bone, cartilage, adipocytes, smooth muscle cells, and other tissues [1]. These tumors are recognized as a particularly intricate domain within diagnostic pathology, presenting complex challenges in both management and diagnosis and exhibiting significant morphological and immune-profile overlaps [2–4]. The 2020 classification by the World Health Organization (WHO) categorizes STTs into eleven groups, which include adipocyte tumors, fibroblastic/myofibroblastic tumors, fibrohistiocytic tumors, vascular tumors, pericytic tumors, smooth muscle tumors, skeletal muscle tumors, gastrointestinal stromal tumors, chondro-osseous tumors, peripheral nerve sheath tumors, tumors of uncertain differentiation, and undifferentiated/unclassified tumors 5. These categories are further classified into four categories on the basis of their biological behavior: malignant tumors, intermediate tumors with rare metastatic potential, locally aggressive intermediate soft tissue tumors, and benign soft tissue tumors [6]. These neoplasms impact various age groups, including adolescents.

The most frequent MTs in children and adolescents are benign and chiefly include vascular tumours and fibrous and fibrohistiocytic tumours [7]. Sarcomas are rare, with an estimated prevalence of 13.7% in adolescents, and represent approximately 7% of all cancers in persons under 20 years of age [8]. In a cohort of young patients presenting with detectable and surface soft tissue masses at a specialized pediatric oncology center, malignant tumors and benign tumors represented 44% and 32%, respectively, while 24% were classified as pseudotumors [9]. The management and outcomes of different entities vary according to their patterns. Hence, biopsy for histological diagnosis is golden, as clinical and radiologic diagnoses are unspecific [7]. 

This study aims to provide a comprehensive clinical and histomorphologic landscape for soft tissue tumours seen among adolescents. This phenomenon is highly important, as health in adolescence sets the stage for health and wellbeing in adult life [10]. 

## Methods

### Study design and setting

This was a cross-sectional study aimed at describing the epidemiology and clinicopathological features of STTs in adolescents who presented to a federal teaching hospital: Nnamdi Azikiwe University Teaching Hospital (NAUTH), Nnewi, between January 01, 2012 and December 31, 2022.

### Data collection

Formalin-fixed and paraffin-embedded (FFPE) tissue blocks of all STTs with confirmed histologic diagnosis in patients aged 10–19 years, were retrieved from the Histopathology Department of NAUTH, Nnewi. Fresh sections were made from the FFPE tissue blocks, stained with H&E, reviewed and reclassified via the 2020 WHO soft tissue tumor categorization.

Clinical data, including sex, age, nature of the specimen, biopsy site, presenting complaints, and duration of illness, were extracted from the request forms and clinical records of the patients.

### Statistical analysis

Statistical analyses were performed using Statistical Package for Social Sciences version 23 (SPSS 23). The age groups were categorized into three as follows: 10–13 years (early adolescence), 14–17 years (mid-adolescence), and 18–19 years (late adolescence). Categorical variables were expressed as frequencies and percentages while numerical variables are expressed as mean and standard deviation. Chi-square analysis was used to test for associations while One-way ANOVA was used to compare categorical data. Significance was set at *p* < 0.05.

### Ethical consideration

This study utilized anonymized archived human tissues and was conducted in accordance with the World Medical Association’s Helsinki Declaration and the National Health Research Ethics Committee guidelines. The study protocol was reviewed and approved by NAUTH Human Research Ethics Committee (NAUTH HREC), Nnewi (Reference: NAUTH/CS/66/VOL.14/VER3/134/2021/036). As the study did not involve direct contact with human subjects, consent to participate was deemed unnecessary by the NAUTH HREC. A hospital permit (Reference: NAUTH/CS/66B/VOL.2/153) was also obtained from NAUTH management before commencement of this study.

## Results

A total of 983 STTs were diagnosed, with 79 being in adolescents; as well as 632 adolescent surgical specimens with histologic diagnoses were seen during the study period. Adolescent STTs accounted for 12.5% of adolescent histologically diagnosed lesions and 8.03% of all STTs. STTs were more (*n* = 28, 35.4%) in mid-adolescents, and largely affected females (*n* = 43; 54.43%) with a male: female ratio of 1:1.2. The mean and median ages of occurrence was 15 ± 2.94 years and 15 years respectively.

Table 1 shows the gender distribution of STTs by age and histogenesis.

**Table 1:** Gender distributions of the STTs by age and WHO categories

Age group (years)	Gender		Total (%)	*p* value
	Male (%)	Female (%)		
10–13	16(61.5)	10(38.5)	26(100.0)	
14–17	15(53.6)	13(46.4)	28(100.0)	0.007*
18–19	5(20.0)	20(80.0)	25(100.0)	
**Total**	36(49.4)	43(50.6)	79(100.0)	
**WHO Categories**				
Skeletal muscle tumours	0 (0.0)	4 (100.0)	4 (100.0)	
Chondro-osseous tumours	5 (55.6)	4 (44.4)	9 (100.0)	
Adipocytic tumours	4 (30.8)	9 (69.2)	13 (100.0)	
Fibroblastic/Myofibroblastic tumours	6 (50.0)	6 (50.0)	12 (100.0)	
So-called Fibrohistiocytic tumours	4(66.7)	2 (33.3)	6 (100.0)	
Smooth muscle tumours	0 (0.0)	1 (100.0)	1 (100.0)	0.303
Vascular tumours	10 (52.6)	9 (47.4)	19 (100.0)	
Peripheral nerve sheath tumours	6 (60.0)	4 (40.0)	10 (100.0)	
Tumours of Uncertain differentiation	0 (0.0)	3 (100.0)	3 (100.0)	
Undifferentiated/unclassified tumours	1 (50.0)	1 (50.0)	2 (100.0)	
**Total**	36 (45.6)	43 (54.4)	79 (100.0)	

Vascular tumours were the most common morphologic category in both genders. No association was found between the WHO categories of STT and gender (*X*^2^ = 10.617; *p* = 0.303), but there was a significant association between sex and the age of occurrence of STTs (χ^2^ = 9.986; *p* = 0.007) (see Table 1).

Table 2 shows the different STTs seen according to the WHO morphologic categories and biologic behaviors.

**Table 2:** The WHO categories of STTs seen in the adolescents

WHO type	Malignant	Intermediate, rarely metastasizing	Intermediate locally aggressive	Benign	Frequency	%
**Adipocytic**					**13**	**16.46**
	Dedifferentiated liposarcoma (*n* = 1)	-	-	Lipoma (*n* = 12)		
**Fibroblastic/Myofibroblastic tumours**					**12**	**15.19**
	Fibrosarcoma (*n* = 2)	DFSP (*n* = 2)	Palmar Fibromatosis (*n* = 1)	Desmoplastic Fibroblastoma (*n* = 3)		
			Desmoid-type fibromatosis (*n* = 3)	Acral fibromyxoma (*n* = 1)		
**So called Fibrohystiocytic tumours**					**6**	**7.59**
				Benign fibrous histiocytoma (*n* = 5)		
				Tenosynovial giant cell tumour, NOS (*n* = 1)		
**Vascular tumours**					**19**	**24.05**
		Kaposi sarcoma (*n* = 2)		Haemangioma (*n* = 15)		
				Cystic lymphangioma (*n* = 1)		
				Lymphangioma, NOS (*N* = 1)		
**Smooth muscle tumours**					**1**	**1.27**
				Pilar leiomyoma (*n* = 1)		
**Skeletal muscle tumours**					**4**	**5.06**
	Embryonal Rhabdomyo (*n* = 3)					
	Alveolar Rhabdomyo (*n* = 1)					
**Peripheral nerve sheath tumours**					**10**	**12.66**
	MPNST (*n* = 5)			Neurofibroma (*n* = 3)		
				Schwannoma (*n* = 1)		
				Triton tumour (*n* = 1)		
**Tumours of Uncertain differentiation**					**3**	**3.80**
	Alveolar Soft Part Sarcoma (*n* = 1)					
	Synovial sarcoma (*n* = 2)					
**Chondro-osseous tumors**					**9**	**11.39**
	Chondrosarcoma (*n* = 2)			Osteochondroma (*n* = 6)		
	Osteosarcoma (*n* = 1)					
**Undifferentiated small round cell sarcoma of bone and soft tissue**					**2**	**2.53**
	Ewing Sarcoma (*n* = 2)					
**Total**	**20**	**4**	**4**	**51**	**79**	**100.0**

The majority (*n* = 51, 64.56%) of the STTs were benign, with hemangiomas being the most common (*n* = 15, 18.99%), followed by lipomas (*n* = 12, 15.19%). Hemangiomas accounted for 78.95% of vascular tumours. Malignant and intermediate-grade neoplasms accounted for 25.32% (*n* = 20) and 10.13% (*n* = 8) of the STTs, respectively. Malignant peripheral nerve sheath tumor (MPNST) was the most common (*n* = 5; 25.00%) malignant STT. Among the cases of MPNST, three occurred in association with neurofibromatosis while one occurred on a background of plexiform neurofibroma. Among the intermediate-grade STTs, desmoid-type fibromatosis was the most common (*n* = 3; 37.50%), whereas Kaposi sarcoma and dermatofibrosarcoma protuberans (DFSP) accounted for 25.0% each. Vascular, adipocytic and fibroblastic/myofibroblastic tumours were the most common histogenic groups, accounting for 24.05%, 16.46% and 15.19%, respectively, of the STTs (see Table 2).

**Fig. 1:**
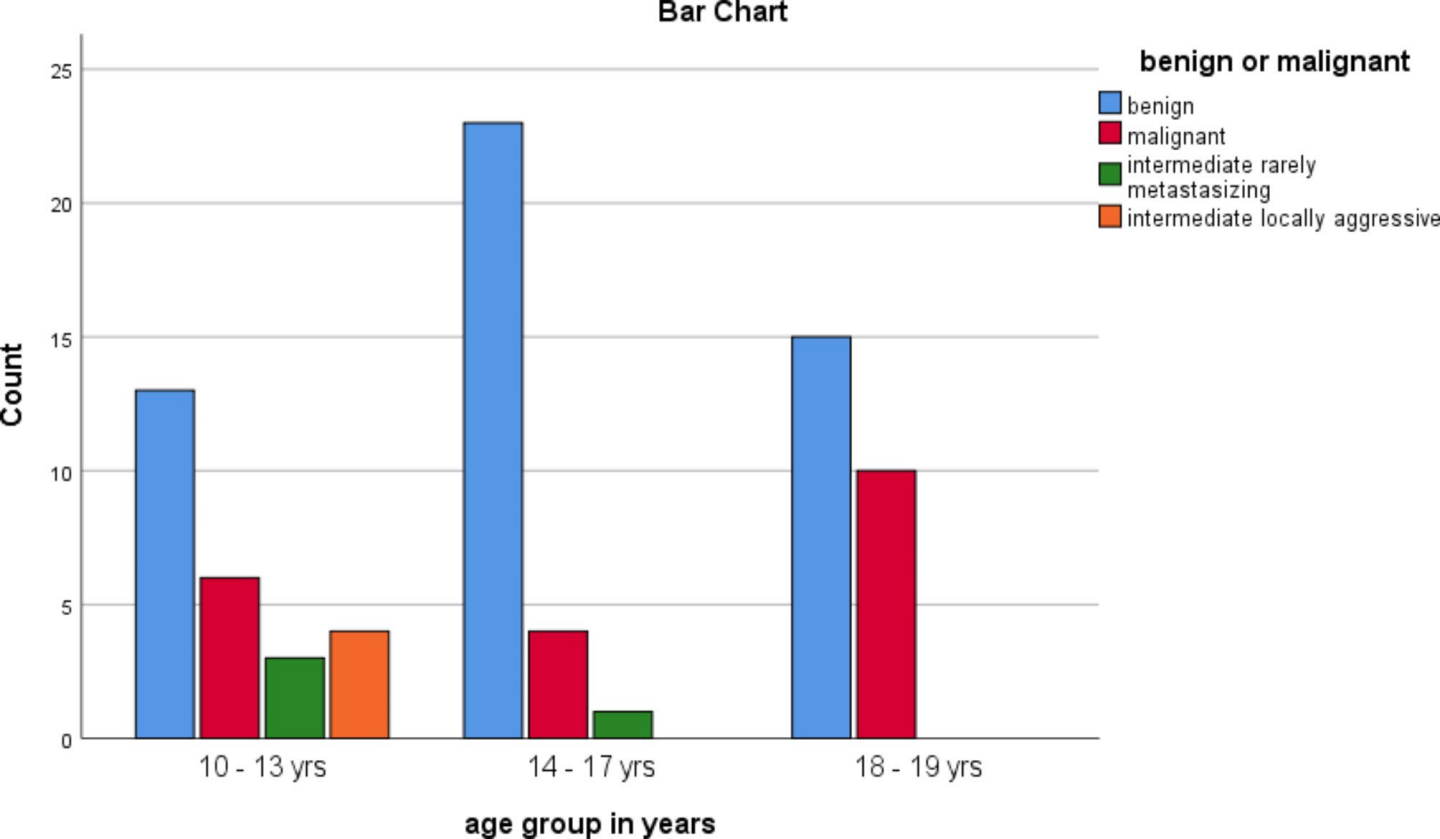
Age distribution of the STT categories by their biologic behaviors

Figure 1 shows the age distribution of the STTs by their biologic behaviors

The mean ages of the patients with benign, intermediate and malignant STTs were 15.20 ± 2.8, 12.00 ± 2.1 and 15.70 ± 2.8 years, respectively. Malignant STTs were more common in late adolescents.

Table 3 shows the relationship between age and the biologic behaviors of the STTs.

**Table 3:** Relationships between age and the biological behavior of patients with STT

Age (yrs)	Biologic behaviour			Total	χ^2^	*p* value	Phi value
	Benign	Malignant	Intermediate				
**10**	5	0	3	8	43.00	0.026*	0.738
**11**	1	1	1	3			
**12**	4	4	0	8			
**13**	3	1	3	7			
**14**	7	1	0	8			
**15**	7	2	0	9			
**16**	4	1	1	6			
**17**	7	1	0	8			
**18**	5	6	0	11			
**19**	8	3	0	11			

There was a significant association between age and the biologic behavior of STTs in adolescents (**χ**^**2**^ = 43.00; *p* = 0.026; phi = 0.738).

Table 4 shows the comparisons of the mean age of occurrence of the different morphologic categories of STTs.

**Table 4:** Mean comparison between age and WHO class of STTs via one-way ANOVA

WHO CATEGORIES	Age Groups			**TOTAL (%)**	**Mean ± STD**	**p value**
	**10–13 yrs (%)**	**14–17 yrs (%)**	**18–19 yrs (%)**			
Skeletal Muscle Tumours	2 (50%)	0 (0%)	2 (50%)	4 (100%)	15.25 ± 3.30	
Chondro-osseous Tumours	1 (11.1%)	4 (44.4%)	4 (44.4%)	9 (100%)	16.00 ± 2.40	
Adipocytic Tumours	3 (23.1%)	4 (30.8%)	6 (46.2%)	13 (100%)	16.15 ± 3.08	
Fibroblastic/Myofibroblastic Tumours	5 (41.7%)	4 (33.3%)	3 (25.0%)	12 (100%)	14.50 ± 2.58	
So-Called Fibrohistiocytic Tumours	2 (33.3%)	3 (50%)	1 (16.7%)	6 (100%)	15.00 ± 3.29	0.191
Smooth Muscle Tumours	1 (100%)	0 (%)	0 (%)	1 (100%)	15.00	
Vascular Tumours	8 (42.1%)	8 (42.1%)	3 (15.8%)	19 (100%)	13.53 ± 3.03	
Peripheral Nerve Sheath Tumours	3 (30.0%)	2 (20.0%)	5 (50.0%)	10 (%)	16.20 ± 2.90	
Tumours Of Uncertain Differentiation	0 (0.0%)	2 (66.7%)	1 (33.3%)	3 (100%)	16.0 ± 2.00	
Undifferentiated/Unclassified Tumour	2 (100%)	0 (0.0%)	0 (0.0%)	2 (100%)	12.5 ± 0.00	
TOTAL	26 (32.9%)	28 (35.4%)	25 (31.6%)	79 (100%)	15 ± 2.94	

ANOVA – Analysis of variance (F = 1.434); *p* = 0.191

The mean age of the different WHO morphologic categories was approximately 16 years, with an F value of 1.434 (*p* = 0.191). There is no statistically significant difference in the mean age across the different WHO morphologic categories.

Table 5 shows the anatomic location and clinical presentation of the STTs.

**Table 5:** Anatomic location and Clinical Presentation of the STTs

Site of biopsy	Frequency	Percent
Head and Neck	20	25.3
Upper limbs	11	13.9
Lower limbs	14	17.7
Back	2	2.5
Chest	8	10.1
Abdomen	15	12.7
Pelvic area	4	5.1
Genitals	3	3.8
Not given	1	1.3
Multiple sites (Chest, Pelvis)	1	1.3
**Total**	79	100.0
**Presenting complaints**		
Painless mass/swelling/growth	66	82.3
Pain	2	2.5
Respiratory distress	2	2.5
Bleeding per vaginam	2	2.5
Painful Mass	4	5.1
No symptom (Incidental finding of mass)	3	1.3
**Total**	79	100.0
**Duration of symptom before diagnosis**		
< 2 years	29	36.7
2–5 years	12	15.2
> 5 years	14	17.7
Not stated	24	30.4
**Total**	79	100.0

The most common presenting symptom was a painless mass (*n* = 66, 82.3%), which commonly involved the head and neck region (*n* = 20, 25.3%). Other accompanying symptoms were weight loss, easy satiety, vomiting, and nipple discharge. Most of the affected adolescents presented within the first 2 years of onset of symptoms (*n* = 29, 36.7%).

Table 6 displays the association between the time duration of illness before presentation and biologic behavior and gender of the STT patients.

**Table 6:** Association between duration of illness and biological behaviour/gender

	Duration of illness				Total	***χ*** ^***2***^	***p***
	< 2 yrs	2–5 yrs	> 5 yrs	Not stated			
Biologic behaviour							
Benign	15	7	12	17	51		
Intermediate	4	2	0	2	8		
Malignant	10	3	2	5	20	10.505	0.105
Total	29	12	14	24	79		
Gender							
Male	17	4	6	9	36		
Female	12	8	8	15	43	3.387	0.336
Total	29	12	14	24	79		
Categories							
Skeletal muscle tumors	4	1	0	0	5		
Bone tumors	4	0	2	0	6		
Adipocytic tumors	1	2	1	2	6		
Fibroblastic/myofibroblastic tumors	2	2	1	7	12		
So-called fibrohystiocytic tumors	0	0	0	1	1		
Smooth muscle	1	0	0	1	2	28.061	0.408
Vascular tumors	6	3	4	6	19		
Peripheral nerve sheath tumors	2	3	3	1	9		
Tumors of uncertain differentiation	4	0	2	3	9		
Uncertain classification	5	1	1	3	10		
Total	29	12	14	24	79		

Table 6 shows that there was no significant association between the biological behavior of STT patients and the duration of illness at presentation (*χ*^2^ = 9.756; *p* = 0.021) or between gender and the duration of illness before presentation (*χ*^2^ = 3.387, *p* = 0.336). Also, no association was found between the histiogenic groups and the period between the onset of symptoms and presentation to the hospital (*χ*^2^ = 28.061; *p* = 0.408).

**Fig. 2:**
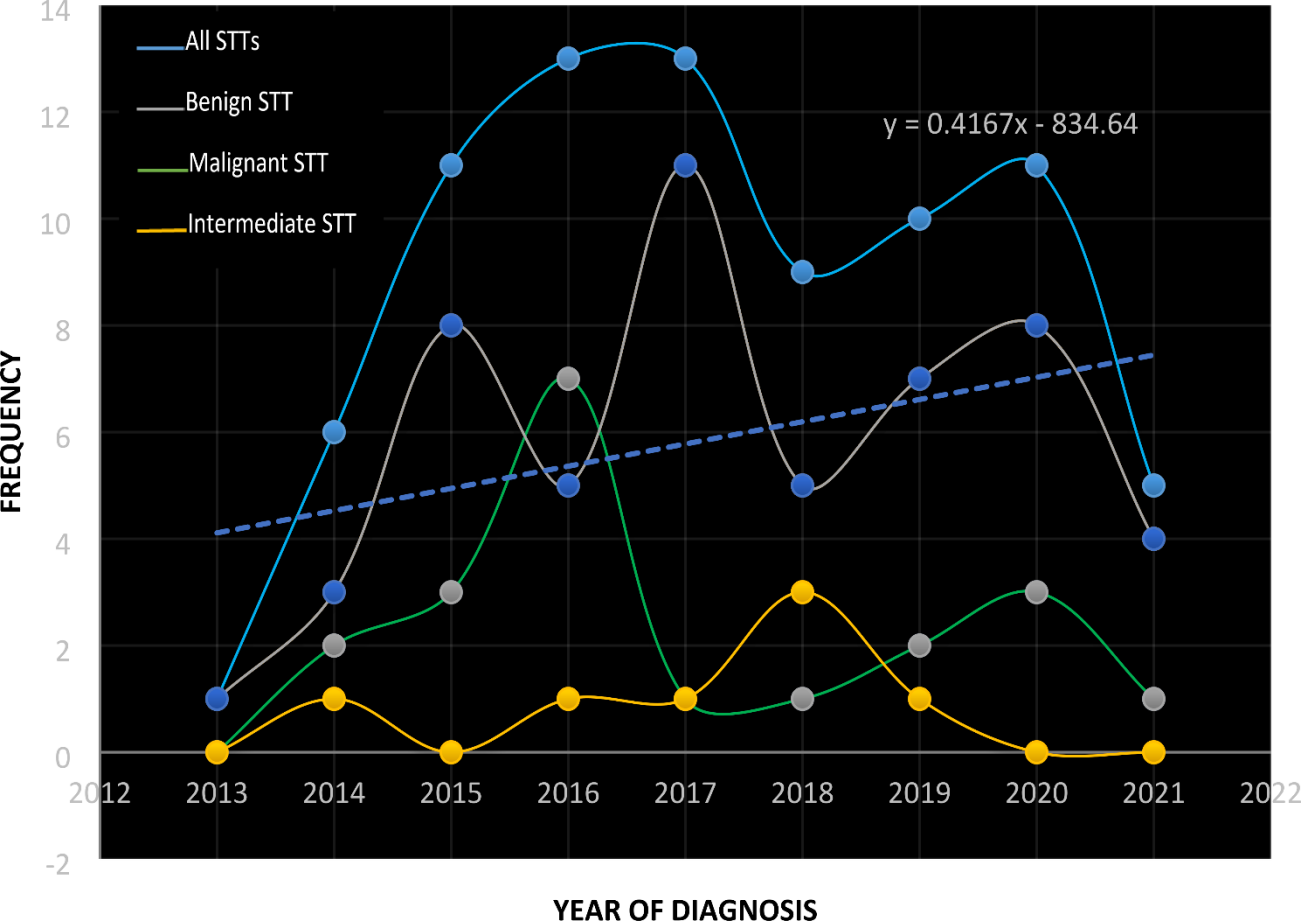
Occurrence of STTs over the study period

Figure 2 shows the occurrence trend for the STTs over the study period. 

There was an increase in the frequency of STTs between 2013 and 206, followed by a decrease thereafter. This decreasing frequency is expected to continue in the next few years (trendline equation is Y = 0.4167x – 831.64).

## Discussion

Adolescent STTs accounted for 12.5% of adolescent histologically diagnosed lesions and 8.03% of all STTs, with 25.32% being malignant. The STTs show a slight female preponderance, with a male: female ratio of 1:1.2, and are more common in middle adolescents. Vascular tumours were the most prevalent STTs in both sexes. There is paucity of studies on adolescent STT in Nigeria and most of Africa. A study done by Odokuma in Benin revealed that adolescent STTs accounted for 81/139 (58.3%) of all childhood STTs [11]. Although a study performed in northern Nigeria did not consider only adolescents, and considered sarcomas, it revealed that adolescent soft tissue sarcomas accounted for 13% of all sarcomas, with rhabdomyosarcoma being the most common (9/16) 12. In a study performed in Bangalore, India, adolescent STT accounted for 12% of all STTs [13]. 

In contrast to our study which revealed a female preponderance, pediatric STTs were reported to be more common in males [11, 14–16]. Although this may point to regional variations in the occurrence of STTs, the difference could be due to the study population (adolescents only) used in our study.

Adolescent STTs present a diverse clinicopathologic spectrum, with soft tissue sarcomas being uncommon [17]. Vascular tumors were the predominant histogenic group in this study, with hemangioma being the most common. These findings agreed with that by Nri-Ezedi et al., which reported that hemangiomas accounted for 17.1% of all benign tumors, being second only to fibroadenomas, but being the predominant soft tissue tumor in the study [18]. However, it contrasted with the findings of a study performed among a pediatric age group in Benin, which reported a predominance of nerve sheath tumours [11]. Other studies performed elsewhere have shown a predominance of adipocytic tumours, although not particularly in adolescents [3, 19–21]. The predominance of vascular tumours in our study may be related to the fact that most of these tumours were managed surgically. Although minimal invasion has been emphasized for the treatment of hemangioma/vascular tumors, these require sophisticated facilities which are not readily available. Hence, most pediatric surgical centers particularly in sub-Saharan Africa, resort to surgical excision. (22–23)

In contrast to our study, which revealed malignant peripheral nerve sheath tumor (MPNST) as the predominant malignant STT, the Benin study reported rhabdomyosarcoma as the most common sarcoma in adolescents [11]. The reason for this is not clear: could it a chance finding or due to locoregional variation? Our observation is that three of the five cases occurred in patients with neurofibromatosis. A recent study in our environment among pediatric patients showed that neurofibroma accounted for 8.1% of benign tumours [18]. Another Nigerian study reported that 17.6% of neurofibroma cases were associated clinically with neurofibromatosis type 1 (NF1) syndrome [24]. Also, MPNST is reported to arise from benign plexiform neurofibroma and borderline atypical neurofibroma in the setting of NF1 [25]. Although there was no case of metastatic STT in our study, Younger et al.. reported that metastatic malignant STTs contribute significantly to disease-related mortality in adolescents, with variable outcomes depending on the histological subtype and treatment modality [17]. 

Most patients presented and were diagnosed within the first two years of illness, and no association was demonstrated either with the biological behavior of the STT or gender. Fairly early presentation facilitates early diagnosis and management. The majority of the patients presented with painless masses, a finding that is in agreement with other reports regarding STT in all age groups [20, 26]. Other features, including bleeding and respiratory distress, could be associated with tumor progression and location [20]. The locations of the STTs are commonly in the head and neck region, and in agreement with the commonly reported locations of hemangiomas [27], which appeared to be the most frequent in this study.

While we observed a significant association between gender and the age of occurrence of STTs (*p* = 0.007), no such association was found between gender and the WHO categories of STTs or between age and the biological behavior of STTs. However, most WHO STT categories in adolescents have a mean age of approximately 16 years. Despite our findings, Tam et al. reported that age-specific molecular profiling is important in identifying risk stratification tools and targeted therapy for adolescents and young adults with STTs [28]. Additionally, the STTs were observed to have a downwards trend since 2020, following a steady increase until 2019. Although the trend equation shows that STT is expected to maintain a downward trend in the coming years, the sharp reduction in 2020 was probably due to the effects of the COVID-19 pandemic on hospital presentations and services [29]. 

## Conclusions

This study revealed that most STTs in adolescents are benign, with vascular tumors being the most common. There was a slight preponderance of females and a decreasing trend in STTs over the study period. No significant association was found between age and gender, but a significant association was found between age and the occurrence of STTs in adolescents. These findings highlight the importance of early diagnosis and management of STTs in adolescents, and inform the development of evidence-based guidelines for the management of STTs in adolescents.

## Data Availability

The dataset on which the conclusions of this study are based will be made available on reasonable request from the corresponding author.
